# Migration Energy Barriers for the Surface and Bulk of Self-Assembly ZnO Nanorods

**DOI:** 10.3390/nano8100811

**Published:** 2018-10-09

**Authors:** Feng-Ming Chang, Zhong-Zhe Wu, Jing-Heng Huang, Wei-Ting Chen, Sanjaya Brahma, Kuang Yao Lo

**Affiliations:** Department of Physics, National Cheng Kung University, Tainan 701, Taiwan; m331529@gmail.com (F.-M.C.); tsungjhewu@gmail.com (Z.-Z.W.); C24021012@gmail.com (J.-H.H.); C24034015@phys.ncku.edu.tw (W.-T.C.); sanjayaphysics@gmail.com (S.B.)

**Keywords:** ZnO nanorods, migration energy barrier, ultraviolet photoemission spectroscopy (UPS), X-ray photoemission spectroscopy (XPS), photoluminescence (PL)

## Abstract

Post-annealing treatment is a necessary process to create/eliminate/repair defects in self–assembly (SA) metal oxide by providing enough thermal energy to the O atoms to overcome the migration energy barrier in ZnO. The height of migration energy barrier is dependent on the depth from the surface, which is hard to be estimated by theoretical calculations, as well as the optical analyses. SA ZnO nanorods (ZNRs) have high surface-to-volume ratio to provide complete picture between the optical and surface properties obtained by photoluminescence (PL) and ultraviolet/X-ray photoemission spectroscopy (UPS/XPS), which is used to investigate the evolution of structure and chemical states of the surface layers to reveal mutual agreement on all observations in PL, XPS, and UPS. We demonstrate variation of the surface structure of SA-ZNRs by scanning over a range of annealing temperatures and time to regulate the structure variation of SA-ZNRs, and their optical analyses agrees well with PL, XPS and UPS, which indicates the dependence of migration energy barriers on the depth from the surface of ZNR. The results reveal the well ZNRs formed at 570 °C and the further oxidation process and the formation of hydroperoxide on the Zn-rich surface of ZNRs at 640 °C.

## 1. Introduction

Metal oxides and their nanostructures are popular subjects because of their applications in photoelectric [1], photo-catalytic [2], and gas/bio sensing [3,4] because of high surface-to-volume ratio that gives rise to typical surface effects. The solution mode growth of metal oxides nanostructures, such as hydrothermal or aqueous solution, provides cheap and convenient method to fabricate nanostructures [5,6]. Defects would be generated during the growth method with quasi-thermal equilibrium [7,8]. Further post-annealing treatments are performed to stabilize nanostructure and eliminate the deficiencies [7,8]. However, the proper annealing temperature and its related treatment on the post-annealing process is not well determined since the energy barriers for eliminating defects on nanostructure should be dependent on the depth from the surface [9]. Especially for the type of self-assembly (SA) materials, several defects are generated during the growth itself [10]. 

The migration energy barrier (*E_b_*) of ZnO is a defined quantity between the equilibrium configuration and the saddle point along the migration path, which can be obtained from first-principles calculations [10]. The knowledge about migration of point defects helps to realize their incorporation during the growth process, impurity diffusion, and modeling self-diffusion [10]. Changes in defects at a given annealing temperature indicate that the relevant defects have become mobile and provides valuable information about the migration energy barrier [10]. The calculated values from the first principle theory can be used to interpret results from self-diffusion measurements and annealing experiments [9]. According to the simulation results of the first principle calculation, the migration energy barriers are dependent on the path and the depth from the surface of ZnO film [11]. Nevertheless, the maximum depth simulated by the first principle calculation only reaches to a few atomic layers [11], which is not a suitable reference to explain the observations obtained from optical analyses of ZnO after post-annealing treatment.

SA-ZnO nanorods (ZNRs) frequently generate many point defects during the growth process and exhibit high levels of unintentional n-type conductivity [12,13], with oxygen vacancies and zinc interstitials being the major composition of native point defects. ZNRs have large surface-to-volume ratio and that provide correlative optical analyses on the surface and bulk parts of ZNRs after post-annealing treatment [14,15,16]. Lorenz et al. elaborated the applications of oxide electronic materials such as optoelectronics, magnetoelectronics, photonics, thermoelectrics, and piezoelectrics. The SA nanocomposites could be fabricated and the surface states could be well-controlled in accurate rate via post-annealing, plasma, and microwave irradiation. The existence of defects, including oxygen vacancies and interstitials, could be inspected by optical measurements, such as photoluminescence (PL) [17]. Pimentel et al. grew ZNRs by the hydrothermal method with microwave irradiation or conventional heating, and then characterized by scanning electron microscope (SEM), PL, Fourier-transform infrared spectroscopy (FTIR), Raman, and X-ray diffraction (XRD) [18]. Araujo et al. conducted the surface enhanced Raman scattering (SERS) measurement on the system of ZNRs with Ag nanoparticles. SERS gives high resolution and exhibits the influence of Ag nanoparticles [19]. However, the progressive migration energy barriers on ZNR surface should be considered to accurately determine the chemical states and optoelectronic properties of annealed ZNRs. Further studies about the migration energy barrier of the point defects in the surface and bulk of ZNRs need systematic and strategic methods to well understand the correlation between the resolution of each optical tools and optical response from ZNRs. The main peak of PL spectra at ~375–380 nm indicates the structural evolution and the broad spectrum in the visible range (400–750 nm) reveals the amount and type of deficiencies in the surface and bulk of ZNRs [20]. Ultra-violet and X-ray photoelectron spectroscopy (UPS and XPS) provide useful surface information (structure evolution and the chemical states) within a thickness of (3–10 nm) [21]. Therefore, the structure characteristics of ZNRs as obtained from PL have considerable correlation with XPS. Based on the correlated response of PL and XPS analyses on ZNRs, it is possible to study the activation energy of ZNR surface by using the annealing treatment with variation in temperature and time. Additionally, traditional O1s XPS analysis on ZNRs focuses on the chemical states of O. It is too complicated to distinguish the contributions from oxygen adsorption, deficiencies and lattice since the binding energy of oxygen on the evolution of restructure and deficiencies would mutually interrupt each other [10]. Furthermore, it can only measure the contributions from oxygen vacancies, but it does not distinguish other different oxygen defects such as oxygen interstitials and oxygen anti-sites. In order to realize the surface structure and chemical states of ZNRs due to the annealing treatment, the chemical states of Zn atoms on the ZNR surface would be a simple inspection to point out the development of ZNR structure [11].

The most challenge on analyzing ZNRs is to separate the optical response from whole ZNR and to realize the corresponding structural variation. In this work, we solved this issue by using a multi-optical method with different depth resolutions to examine annealed ZNRs whose structural evolution was well-controlled with the method of scanning annealing temperature and time. The evolution of structure and deficiencies would occur as annealing temperature crossover the threshold of migration energy barrier which is dependent on the depth from the surface. The surface activation energies of restructure and deficiencies elimination for ZNRs are investigated by using multi-optical way with PL, XPS, and UPS. The systematic analyses on a series of annealed ZNRs examine the correlation between the optical analyses and the annealing temperature (time) in an interval. The purpose of the designed processes is to build the analysis method with scanning annealing temperature and time to approach migration energy barriers on ZNR surface. Additionally, more complicated chemical states of Zn on the shallow surface of ZNRs obtained by XPS and UPS is discussed in more detail.

## 2. Materials and Methods 

The zinc oxide seed layers were deposited on glass substrate by the RF sputtering with 99.99% ZnO target. ZNRs were grown on the ZnO seed layer with hydrothermal method with aqueous solution of 25 mM zinc nitrate hexahydrate (Zn(NO_3_)_2_·6H_2_O, 99.0%) and 25 mM hexamethylenetetramine (C_6_H_12_N_4_, 99.0%) (CHONEYE Co., LTD., Taiwan). The ZnO seed layer was immersed in the aqueous solution at 90 °C for 8 h in hot-air oven (DENGYNG Co., Ltd., Taiwan) to grow the ZNRs [3]. 

The rapid thermal annealing (RTA) system (SHIHSIN Technology Co., Ltd., Taiwan) was applied in this work to perform the structural variation of ZNRs. The temperature increasing rate was 45 °C per second. The annealing temperature and duration time in the RTA system can be well controlled. The annealing process in the RTA chamber was conducted under the pressure of 2 × 10^−6^ torr.

The ZNRs were annealed at different temperature (from 300 °C to 700 °C) with several annealing duration (10 s/30 s/60 s). For convenience, the annealing conditions were labeled in the form of (temperature) or (temperature, annealing duration), for instance, (300,10) represent annealing at 300 °C for 10 s. The photoluminescence (PL) spectrum was characterized by using He-Cd laser (wavelength is 325 nm) (KIMMON KOHA Co., Ltd., Japan) as excitation source. The X-ray and Ultraviolet photoemission spectroscopy (XPS and UPS) were conducted at BL24A, NSRRC. The photon energy for O1s XPS and Zn2p XPS were 625 and 1100 eV, respectively, and the results of XPS were calibrated with the peak of C1s (284.8 eV). The photon energy for UPS is 40 eV and the results of UPS were calibrated against Au4f 7/2 (83.9 eV).

## 3. Results and Discussion

### 3.1. Morphology Characterization

**Figure 1:**
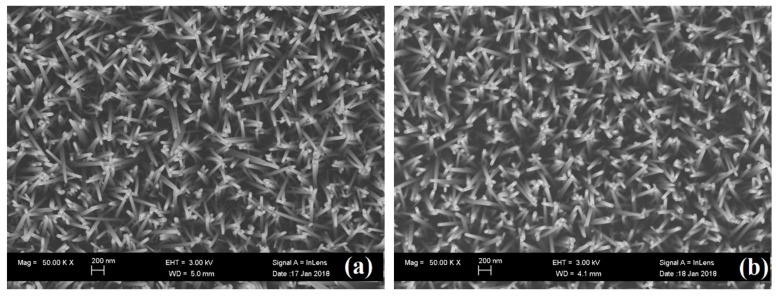
SEM images of (**a**) As growth ZNRs and (**b**) ZNRs annealed with condition of (660,30).

In order to realize the variation of morphology of ZNRs via annealing, the observation of SEM images was performed on these ZNRs. SEM image of as growth ZNRs and ZNRs annealed with (660,30) are shown in Figure 1a,b, respectively. ZNRs are uniform distribution with the diameter of ~50 nm and the aspect ratio of ~20. The morphology and distribution of ZNRs kept invariant after annealing at high temperature (660 °C).

### 3.2. Evolution in PL Spectra

**Figure 2:**
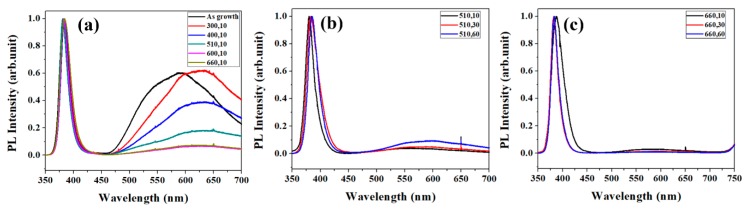
(**a**) PL for varied annealing temperature with the time of 10 s. (**b**) PL for the annealing temperature of 510 °C with varied time. (**c**) PL for the annealing temperature of 660 °C with varied time.

Figure 2a shows the PL spectra for ZNRs annealed at different annealing temperature keeping same annealing time (10 s). The as prepared ZNRs show a strong visible emission peak centered at ~575 nm and the peak shifts to higher wavelength (630–640 nm) after annealing at 300 °C. Further high temperature annealing from 400–660 °C leads to decrease in the intensity of the peak. Although the intensity of the visible luminescence peak of as prepared and 300 °C is nearly same, the peak shift indicates the difference in the type of defects in these two samples. The deficiencies in ZNRs increase and the structure of ZNRs is deteriorated at lower annealing temperature (300 °C and 400 °C), which is reflected by the broad band in the visible region of PL and wider full width at half maximum (FWHM) of the main peak. The results agree with earlier reports where OH^-^ bonding in SA-ZNRs cannot be eliminated at the annealing temperature below 400 °C [22]. As the annealing temperature increases, the peak of ZnO band gap (around 400 nm) shifts to the shortest wavelength and induces narrowest FWHM at (510,10), and the visible-light region of PL is also nearly suppressed. As annealing time is increased at 510 °C, the structure of ZNRs is getting worse and more deficiencies are generated in ZNRs gradually, as shown in Figure 2b. That means the annealing treatment at 510 °C can only recover the top surface of ZNRs and the 510 °C is too low to activate the deep layers (bulk) of ZNRs. The annealing treatment at 510 °C is enough to activate the top surface layers of ZNRs, but cannot still overcome the migration energy barrier at deeper layers (ZNR bulk). Additionally, more defects in the bulk of ZNR bulk are formed at lower temperature. Figure 2c shows the PL spectra of ZNRs annealed at the temperature of 660 °C. The main peak shifted to shorter wavelength and FWHM of the main peak decreases with the increase of the annealing time. Meanwhile, the visible light region of PL is progressively suppressed. The annealing treatment at 660 °C should overcome the migration energy barrier of the ZNR bulk and eliminate deficiencies in ZNRs, and, therefore, the visible light region in PL keeps similar low intensity and is not influenced by the annealing time.

### 3.3. Evolution in XPS 

#### 3.3.1. Complicated Progression of Deficiencies Analyzed by O1s XPS

**Figure 3:**
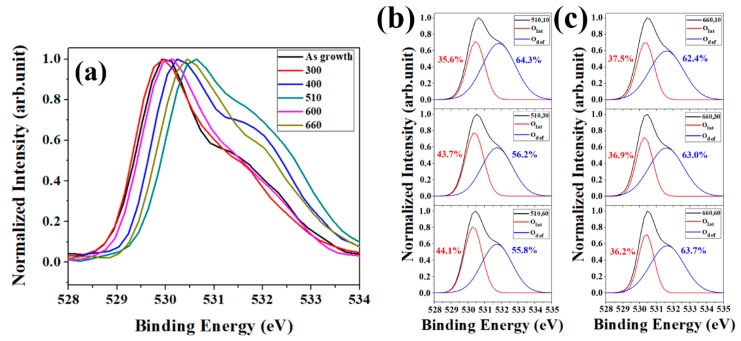
(**a**) O1s XPS for varied annealing temperature with the time of 10 s. (**b**) O1s XPS for the annealing temperature of 510 °C with varied time. (**c**) O1s XPS for the annealing temperature of 660 °C with varied time.

Deficiencies and restructure behavior of ZNRs via thermal annealing would reflect in the PL spectra, where the contribution of the main peak in the visible light region dominate from the surface layers of ZNRs due to the high surface-to-volume ratio of ZNRs. O1s XPS on annealed ZNRs with annealing time is a possible way to analyze the surface deficiencies since the resolution of XPS is about 5–10 nm. Figure 3 shows the O1s XPS for different annealing temperatures and at same annealing time of 10 s. The variation of O1s XPS involves the complicated behaviors of O in the top surface of ZNRs. The peak of binding energy shifts toward 530 eV from the as-grown ZNRs to the annealing temperature of 400 °C, which revealed the release of OH^−^ in ZNRs (i.e., the existence of OH^−^ would decrease the binding energy due to the oxidation numbers in ZnO reduces). As the annealing temperature increases to 510 °C, the profile of O1s XPS shifts to lower binding energy due to the increase of the oxygen vacancies in ZNRs (531 eV) and oxygen is adsorbed on the top surface of ZNRs (at 532 eV). As the annealing temperature increases to 600 °C, the surface oxygen vacancies are further eliminated and the binding energy of O1s returns back to 530 eV. Further increase in the annealing temperature to 600 °C, XPS pattern mostly occupied on the binding energy among 531 and 532 eV. More deficiencies were generated at the higher annealing temperature (660 °C) and then broaden the FWHM of XPS pattern, which caused by the migration/adsorption of oxygen atoms over the surface layer [23]. However, deficiencies such as V_o_, O_i_, and O_Zn_ could not be distinguished at the binding energy of ~532 eV and the oxygen chemical adsorption (O_ads_) would also be measured at ~532 eV of binding energy. O1s XPS can inspect the evolution of deficiencies in the ZnO matrix via annealing treatment, but it is hard to realize the surface activation of ZNRs since there are many complicated phenomena among ZnO matrix, oxygen adsorption, and deficiencies during annealing [24]. In order to examine the correlation between the migration energy barrier and the depth for oxide nanostructure, the exhibition of Figure 3b,c would be the well interpretation of the structural evolution of ZNRs at different annealing temperature and times. The peak area ratio at 530 eV in XPS (A_ZnO_: the characteristics of ZnO matrix) gradually increases with the annealing time at the annealing temperature of 510 °C and that remains almost unchanged at (660,10), (660,30), and (660,60). The results reveal the deficiencies in the surface layer of ZNRs are eliminated in 10 s since the energy barrier of the surface overcome at (660, 10). 

#### 3.3.2. Identify ZnO Chemical States from Zn2p XPS

**Figure 4:**
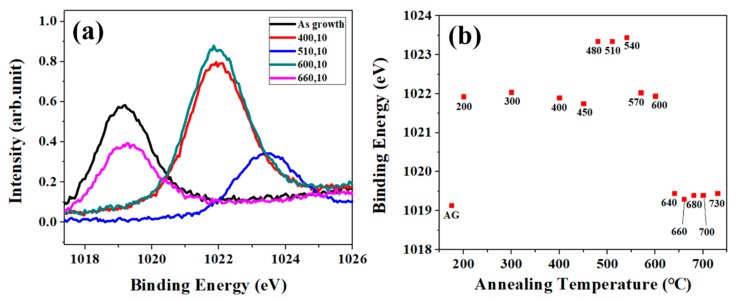
(**a**) Zn2p XPS for SA-ZNRs and ZNRs annealed at varied temperature. (**b**) The peak shift of Zn2p in XPS.

The relative ratio of the peak area for each specific binding energy in O1s XPS is not easy to correlate the evolution of chemical states of ZNRs via annealing. Although, the evolution of *A_ZnO_* in O1s XPS for varied annealing condition is considerable, the changes in Zn2p XPS can be treated as the precise index for the structure development in ZnO matrix via annealing. Figure 4a shows Zn2p XPS for as-grown ZNRs and ZNRs annealed at varied temperature with a fixed annealing time of 10 s and Figure 4b shows the corresponding binding energy for as-grown ZNRs and annealed ZNRs as obtained in Figure 4a. We use the shift of Zn 2p binding energy to replace the comparison between the peak area of Zn element (1021.7 eV) and ZnO lattice (1022 eV), which clarifies the evolution of the chemical states of Zn at different annealing temperature. There are three progressions in Zn2p from the condition of as growth to 730 °C. It is reasonable to analyze the development of Zn2p XPS by referring to successive PL spectra in Figure 2. As the annealing temperature is below 450 °C, the structure of ZNRs is worse since the energy gap of ZNRs is getting narrower and FWHM of main peak in PL is wider due to the incorporation of OH^−^ in ZNRs. However, the binding energy of Zn belongs to the characteristics of ZnO lattice. The increase in the binding energy of Zn2p indicates that the stoichiometry of ZNR matrix is getting better with the increase of the annealing temperature to 510 °C, and this temperature provides enough activation energy to the shallow surface layer, but deeper surface layers (ZNR bulk) need larger activation energies to overcome the migration barrier. As the annealing temperature increases to 660 °C, the binding energy of Zn2p in XPS decreases and belongs to the characteristics of Zn element. As same explanation in the PL spectra for the condition of 660 °C, the provided energy budge is high enough to overcome the migration energy barriers in deeper surface layers (ZNR bulk). Moreover, the high thermal budge causes the evaporation/dissolution of bound oxygen from the top layer of ZnO matrix, which generates additional deficiencies on the top surface layers.

### 3.4. Evolution in UPS 

**Figure 5:**
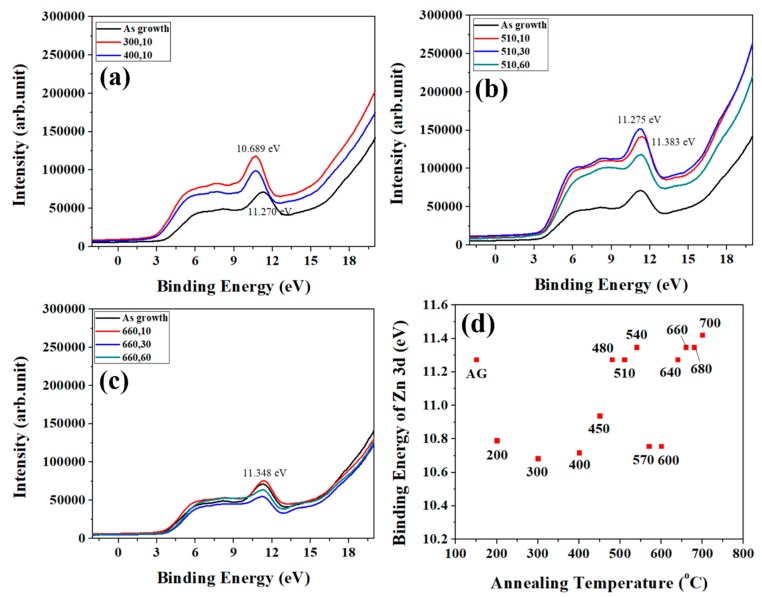
(**a**) UPS for annealing temperature of 300 and 400 °C. (**b**) UPS for annealing temperature of 510 °C with varied time. (**c**) UPS for annealing temperature of 660 °C with varied time. UPS for as-grown ZNRs is a reference in each diagram. (**d**) The trend of the binding energy of Zn3d for varied annealing temperature at 10 s.

In order to analyze the top surface layer of ZNRs more particularly, UPS experiments with synchrotron radiation source are performed on as-grown and annealed (300 °C and 400 °C) samples as shown in Figure 5a. As the thermal energy provided from the annealing temperature of 510 and 660 °C excess the migration energy barrier in the depth of 3 nm (the depth of resolution in UPS), there is no distinction in the pattern for annealing time 10 s and 60 s, as shown in Figure 5b,c, respectively. Figure 5d indicates the binding energy of Zn3d in UPS for different annealing temperature with 10 s. There are similar trends in UPS (Figure 5d) and XPS (Figure 4b), which have four sections and same turning temperature on both trend analyses. However, the as-grown and the last section in UPS have some discrepancies with the results of XPS.

### 3.5. The Similarities and Dissimilarities Between XPS and UPS on the Analyses of ZNRs

**Figure 6:**
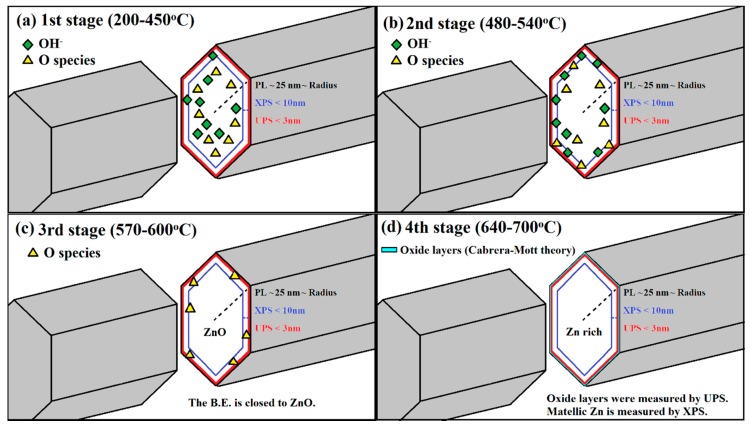
The similarities and dissimilarities between XPS and UPS (**a**) the first stage (200–450 °C): the surface adsorption on ZNRs was eliminated. (**b**) The second stage (480–540 °C): OH^−^ diffuse and oxygen slower migrate to the surface of ZNRs. (**c**) The third stage (570–600 °C): remained migrated-oxygen atoms still halted on the top surface of completed ZNRs. (**d**) The fourth stage (640–700 °C): oxidation process occurred at the top surface layer of Zn-rich ZNRs.

Results of Zn2p XPS and Zn3d UPS have some discrepancies on the evolution of the binding energy of Zn via annealing. The analysis method of XPS and UPS on the structural evolution of ZNRs is based on some hypotheses. (1) OH^−^ and oxygen atoms out-diffuse during annealing; (2) the resolution of Zn2p XPS and Zn3d UPS is 2 nm and 8 nm, respectively, which is dependent on the photon energy of synchrotron light [18]; and (3) the annealing time for varied annealing temperature is maintained at 10 s to normalize the amount of jumped atoms. The phenomena for each stage in Zn2p XPS and Zn3d UPS are explained as below:

(1) In the first stage (200–450 °C), as diagrammed in Figure 6a: the elimination of the surface adsorption and the migration behavior of atoms initially occurred. The binding energy of Zn decreases as shown in UPS due to the elimination of surface adsorption on ZNRs. The initialization of the migration effect is revealed in XPS and the binding energy of Zn shifts from the Zn metal state (101.9 eV) to the ZnO state (1022 eV). As OH^-^ or oxygen atoms migrated to the surface within the resolution range of XPS, the binding energy of Zn trends to the characteristics of ZnO state. As several oxygen layers adsorbed on the top surface of as-grown ZNRs, this result leads to the increase of the binding energy of Zn (the oxidation numbers) on the top surface. The resolution depth of UPS is ultra-shallow. Therefore, the signal integration of UPS usually focus on the top surface layers and UPS exhibits the result of higher oxidation numbers.
(2) In the second stage (480–540 °C), as diagrammed in Figure 6b: OH^−^ diffused and oxygen migrates slowly to the ZNR surface, the binding energy of Zn in XPS and UPS reached to the higher value due to the increase of the oxidation numbers. The process of OH^-^ out-diffusion has happend at this stage.
(3) In the third stage (570–600 °C), as diagrammed in Figure 6c: All OH^−^ left the ZNR surface, and the remaining oxygen atoms still exist on the top of ZNR surface. This leads to the decrease of the binding energy of Zn2p in XPS and Zn3d in UPS.
(4) In the last stage (640–700 °C), as diagrammed in Figure 6d: oxygen atoms over the surface of ZnO matrix are evaporated and ZnO matrix lose oxygen atoms with the reduction of the oxidation numbers and the binding energy of Zn2p in XPS is decreased. Interestingly, the binding energy of Zn3d in UPS is increased as the binding energy of O1s in XPS is blue shifted. Oxygen atoms evaporated from the surface of ZNRs leaves metallic Zn on the top surface. As the ZNR samples are exposed to the air before XPS and UPS experiments, the oxidization process would occur on metallic Zn atoms of ZNR surface according to the Cabrera-Mott theory [25]. Oxygen molecules are adsorbed on metallic Zn of ZNR surface and then acquire electrons to form ionized oxygen. Sequentially, two or three oxide layers are formed till an electrical field is generated between the metallic Zn and the oxide layer, which suppresses further oxidization [26]. In the Zn enrichment surface, the additional electrical potential on the top two-three layers of ZNR surface caused by the oxidization process and the hydroperoxide (ZnO_2_) may form on the top layer due to adsorption of more oxygen on the top surface of ZNRs [22]. It is why the binding energy of Zn3d is getting larger in this stage, which has larger discrepancies with XPS due to the large difference in the depth of resolution. The UPS is sensitive to the top surface of ZNR. Unavoidably, the Zn3d in UPS reveals this phenomenon which agrees with the oxygen behavior in the results O1s in XPS.

### 3.6. Mechanism of Migration Energy Barriers and Defect Migration in ZNRs 

**Figure 7:**
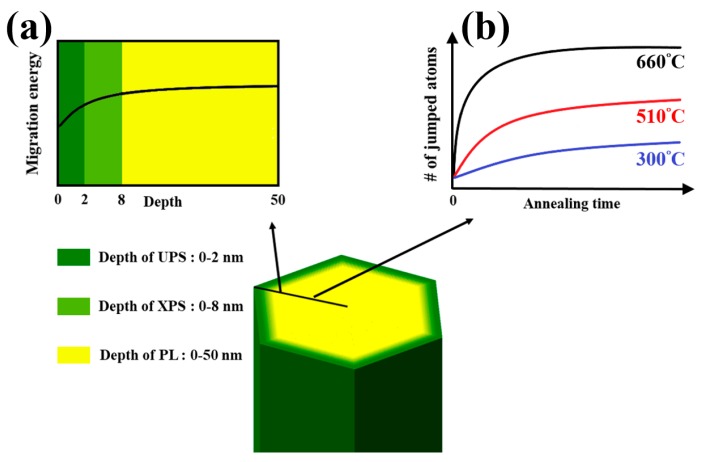
The distribution of the migration energy barrier in ZNRs (**a**) The trend of the surface migration energy barriers in the depth of 50 nm from the ZNR surface. (**b**) The correlation of annealing temperature and time to reach the completed ZNRs.

From the experimental results and the development on the optical properties, the energy of eliminating defects increases from the surface to bulk of ZNRs. Tuning on the annealing temperature and time can identify the species and amount of defects on the top surface layer or bulk of ZNRs via the analyses of multi-optical method. According to the transition state theory [10,27], defects in ZNRs are eliminated through an atom near a vacancy jumps to this vacancy (or an interstitial jumps to the near interstitial site) while atoms or interstitials obtain enough thermal energy to overcome the migration energy barrier. The annealing treatment provides additional thermal energy to raise the jump rate. The jump frequency (rate) of defect migration is expressed as:

(1) $$\Gamma =\Gamma_{0}exp\left(\frac{-E_{b}}{k_{B}T}\right)$$

where $\Gamma_{0}$ is the ratio of the vibrational frequencies at the initial configuration to the frequencies at the saddle point, *k_B_* is the Boltzmann constant, and *T* is the annealing temperature. The corresponding activation energy for migration barrier can be estimated by the annealing temperature [10,28]. Above experimental results indicate that the oxygen deficiencies in the surface and bulk are eliminated as the annealing temperature is above 660 °C, which agrees well with the previous theoretical model [10,28]. The defects in the surface can be eliminated at the annealing temperature of 510 °C and the estimated *E_b_* is about 2.03 eV. The migration energy barrier of the ZNR surface is obviously lower than the one of ZNR bulk. The schematic of the phenomenon and mechanism of migration energy barriers are shown in Figure 7. The jumped atoms should have enough energy to overcome the migration barriers of ZNR surface, the response of PL, XPS and UPS reveal strong dependence on the annealing temperature. The trend of the surface migration energy barriers in the depth of 50 nm from the ZNR surface is shown in Figure 7a. The value of migration energy barrier gradually increases from the surface and that remains constant in the bulk. UPS and XPS can inspect the variation of chemical states of Zn at the surface layer of 2 nm and 8 nm, respectively. PL measured the whole volume of ZNRs (the diameter of 50 nm). The results of UPS and XPS is comparable to PL since the surface analyzed volume of UPS and XPS is 10% and 30% of whole ZNR. The migration energy barrier is dependent on the depth from the surface, the optical response would reflect to the condition with varied annealing time if the annealing temperature below the threshold annealing temperature. Though the jump rate is lower in the annealing temperature at 510 °C, longer annealing time provides more jumped atoms to eliminate defects gradually, as shown in PL, XPS, and UPS. However, the annealing treatment at higher temperature (i.e., the annealing temperature >640 °C) can eliminate defects in short time, even approaching to the ZNR bulk. Figure 7b diagrammed the correlation between annealing temperature and time. At low annealing temperature, the number of jumped atoms are still low irrespective of the duration of annealing time since only few atoms cross the energy barriers. Conversely, the defect elimination is high at high annealing temperature, and that saturates within a short time. The thermal energy of atoms is enough to overcome the migration energy barrier of the ZNR surface (i.e., the annealing temperature > 640 °C), the results of XPS and UPS remains unchanged at high annealing temperature. Besides, the defects of V_o_^+^ (1.91 eV) [9] would be formed in the ZNR bulk as observed in PL with the condition of (510,30) and (510,60). The post-annealing treatment at lower temperature can repair the deficiencies in the ZNR surface, but the more deficiencies (such as the species of V_o_^+^) would be formed at this temperature.

Some of the existence bottlenecks on analyzing ZNRs are to separate the optical response of ZNR surface from whole ZNR and controllable modification on the structural variation of ZNR surface. In order to realize the structural evolution and migration energy barriers of ZNR surface, the methods of multi-optical analyses with distinguishable depth resolution and post-treatment with precisely controlling annealing conditions are provided to overcome the bottlenecks. Additionally, the knowledge about the surface migration energy barriers on ZNRs is helpful for design ZNR sensors [29] whose sensitivity is strongly dependent on the configuration of ZNR surface.

## 4. Conclusions

Above inspections on PL, XPS, and UPS for annealed SA-ZNRs with scanning temperature provide the quantitative analyses on the restructure and annihilation of deficiencies. As the discrepancy on PL and XPS, the structure evolution in depth can be inspected and reveal activation energies with the dependence of depth. The restructure and the elimination of deficiencies of SA-ZNRs occur in the limited depth if the annealing temperature is limited within 470–540 °C even at longer annealing time. It is obvious that the annealing treatment with specific temperature can only restructure the SA-ZNRs with certain depth which determines the corresponding activation energy. The amplitude of activation energy is certainly correlated to the depth of ZNRs. Moreover, the surface layers of SA-ZNRs were not only restructured but also create another set of deficiencies according to the annealing environment if the annealing process lasts longer time at higher temperature. The further application based on the knowledge about the evolution of the migration energy barriers on ZNR surface would improve the fabrication of ZNR sensors [29]. The sensitivity of ZNRs is strongly correlated with the quality and chemical states of ZNRs surface. To understand the surface migration energy barriers of ZNRs well, the surface configuration of ZNRs should be controlled well by precise post-annealing to develop ZNR sensors with high sensitivity.
